# Study of CFU for individual microorganisms in mixed cultures with a known ratio using MBRT

**DOI:** 10.1186/s13568-014-0038-7

**Published:** 2014-04-05

**Authors:** Subir Kumar Nandy, KV Venkatesh

**Affiliations:** 1 Department of Chemical Engineering, Indian Institute of Technology, Bombay, Powai 400 076, Mumbai, India

**Keywords:** Colony forming units (CFU), metabolic active cell, Methylene blue dye, Escherichia coli, Bacillus subtilis

## Abstract

Determination of metabolically active cell count is an important step in designing, operating and controlling fermentation processes. It’s particularly relevant in processes involving mixed cultures, where multiple species contribute to the total growth. The motivation for the current study is to develop a methodology to estimate metabolically active cell counts for the individual species in a mixed culture with approximate equal numbers. Further, the methodology should indicate the presence of a contaminant in short time periods since in the agar plate methods used frequently it takes about 24 h. We present a methodology based on the rate of Methylene blue (MB) reduction to evaluate total count of metabolically active cells. The standard curve relating the slope of MB reduction and CFU of the individual species could be used to measure the metabolic activity of each species in the mixed culture. The slope of MB reduction could also be used to obtain the growth rate of individual species in a mixed culture and that of the total cell count. These measurements were achieved in less than 6 minutes during the growth of the cells. Evaluating the metabolic activity of individual species in a mixed culture is tedious, difficult and time consuming. The Methylene Blue dye Reduction Test (MBRT) presented here is capable of quickly estimating colony forming units (CFU) of individual species in a mixed culture if the ratio of the numbers of cells is known. The method was used to dynamically detect the occurrence of a contaminating microorganism during fermentation. The protocol developed here can be adapted to applications in processes involving mixed cultures.

## Introduction

Quantification of metabolic active cell is essential for design, operation and control of processes involving mixed cultures. Processes involving mixed cultures include biological waste treatment and food processing. Several methods have been used in the past to measure CFU including agar plating, imaging using microscope and fluorescence techniques, membrane filtration and flow cytometry. But, most of these methods are labor intensive, error prone, time consuming, instrumentation intensive and expensive (Auty et al. [2001], Davey and Kell [1996], Walley and Germida [1995], Wright et al. [1993]).

Thus, there is a need for fast and accurate method to enumerate metabolic active cell count of organisms present in a mixed culture. Metabolic active cell of pure culture is typically quantified using plate count assay such as streak and spread plate methods. However, these methods are time consuming although they are cheap and easy to perform. Enzyme activity specific to an organism has also been used to quantify metabolic active cell (Walley and Germida [1995], Callaway et al. [2006]). Imaging is another common technique that is used to evaluate metabolic active cell. This is achieved by staining the cells using acridine orange, propidium iodide or rhodamine (Wright et al. [1993], Miller et al. [1993]). Confocal scanning laser microscopy (CSLM) is a microscopic technique to determine metabolic active cell and is an accurate method (Schmidt et al. [2007]). However, the method is expensive and requires operation and availability of the confocal microscope. For metabolic active count of cells in liquid medium, epifluorescence microscopy has been used in the past (Vaija et al. [1993]). Flow cytometry is also a technique that yields accurate cell counts (Davey and Kell [1996], Deere et al. [1998]). The above methods are instrument dependent and expensive. The methods involving dyes are error prone as it is difficult to differentiate live from dead cells (Schmidt et al. [2007]). Recently, our group has developed a protocol for fast estimation of cell count using the Methylene Blue Dye Reduction Test (MBRT) (Bapat et al. [2006]). Methylene blue, a dye, is reduced by transmembrane reductases present on the cell membrane, which reduces methylene blue (MB) and thus the blue color disappears. The rate of disappearance of the color is correlated to the cell count of any aerobic organisms. This method can be used to obtain colony forming units in about 3 minutes (Bapat et al. [2006]).

Although various methods exist for enumerating colony forming units or metabolic active cell for pure cultures, it is a difficult task to quantify metabolic active cell in a mixed culture. Since the individual metabolic active cell needs to be enumerated along with the total metabolic cell activity of a mixed culture. Agar plating using differential agar plates for specific organisms is commonly used to quantify metabolic active cell in mixed cultures. For example, MacConkey agar plates are commonly used to differentiate the number of *Escherichia coli* cells in a mixed culture (Fenlon and Wilson [2000]). The bile salts and crystal violet dye presents in MacConkey agar inhibits the gram positive bacteria such as *Bacillus subtilis*. On the other hand phenyl ethyl alcohol agar plates are used to estimate gram positive organisms such as *B. subtilis* (DuTeau et al. [1998]). Enzyme linked immunosorbant assay can be used to quantify specific organism in a biofilm or mixed cultures (Bauer-Kreisel et al. [1996], David et al. [1995]). Terminal-Restriction fragment length polymorphism has also been used to specifically quantify cells in cultures containing more than two cultures (Schmidt et al. [2007]). Real time PCR based method has been used to enumerate *Listeria monocytogenes* in food samples at very high sensitivity (Schmidt et al. [2007]). A laser integrated microarray scanner was used to quantify and compare the biomass of *Burkholderia cepacia* G4 alone in presence of phenol degrading community of microorganisms (Callister et al. [2003]). This method could detect upto 10^3^-10^4^ cells ml^−1^. Hence, the methods are either expensive and accurate or cheap and error prone.

Here, we extend the MBRT to quantify metabolic active cells in mixed cultures. The method yields the total metabolic active cell and CFU of individual cells present in the mixed cultures. We have developed and standardized the method by monitoring and relating the dye reduction rate to the metabolic active cell count of *E. coli* and *B. Subtilis*. The MBRT test correlates with Colony Forming Units (CFU) up to a 1000 live cells as established by plating. Developed MBRT test is very simple, fast (200 s) and inexpensive as compared to available techniques. The protocol was also used to predict the presence of a contaminant during growth of pure culture.

## Materials and methods

### Microorganisms

*Escherichia coli* K12 (MTCC 1302) and *Bacillus subtilis*168trpC2 (Nandy et al. [2007]) were used throughout the study. The *E. coli* K12 strain was obtained from MTCC, IMTECH Chandigarh, India and *B. subtilis* 168trpC2 was a gift from the lab of Prof. K.K. Rao, School of Biosciences and Bioengineering, IIT Bombay. Both of the strains were maintained on Luria broth agar slants. A loopful of the culture from the slant was subculture before each test and transferred into 100 ml of sterile Luria broth (Hi-media, Mumbai, India; Cat. No. M1245) and grown for 6 h at 37°C at 240 rpm. The 10% (v/v) of this seed was then added to 100 ml sterile Luria broth and grown for another 24 to 36 h for kinetic analysis.

### Chemicals

Luria broth, Luria agar (LA), MacConkey agar and Phenyl ethyl Alcohol Hiveg™ Agar (Hi-media, Mumbai, India) were used throughout the experiments to grow the organism, maintain culture on slant and for obtaining metabolic active cell count using spread plate method, respectively. Methylene blue dye was obtained from E. Merck, India.

### Methylene blue dye reduction test

Methylene blue dye was prepared by dissolving 1 g of the dye in 100 ml double distilled water. This 1% dye was used throughout the study. Spectrophotometer was used to obtain the rate of disappearance of blue color at 700 nm for 200 s. Cuvettes containing metabolic active cells were used to get the rate of decoloration. Methylene blue is reduced by respiratory cells, and thus the rate of decoloration could be correlated to metabolically active cell counts.

During the time course measurement in a spectrophotometer, cuvettes were covered with a plastic lid to prevent fresh oxygen dissolution into the culture. In the dye reduction test dead bacterial cells were used as a negative control. The quartz cuvettes were used instead of plastic to minimize error caused by the adhesion of methylene blue dye over a period of time. Further, the MB dye concentration and the blue color intensity was also linear in the range of 0.1-1.0 g l^−1^ of methylene blue indicating that any concentration in this range can be used for the assay (Bapat et al. [2006]).

*E. coli* and *B. subtilis* were grown separately for 8 h in shake flasks at 240 rpm and at 37°C. The whole broth was centrifuged at 10,000 rpm (Remi C-24 centrifuge, India) (after 6–8 h of incubation). Cells from each culture flasks were resuspended in 10 ml PBS of pH 8. The cells were further either diluted or concentrated to yield various concentrations of initial cell number in PBS. The cell number varied from 10^14^-10^2^ cells/ml of both *E. coli* and *B. subtilis*. The test-tubes containing equal number of *E. coli* and *B. subtilis* were mixed in equal quantity (5 ml each) to obtain mixed culture of the two strains containing equal number of the metabolic active cells. The sample containing the highest cell number (i.e. 10^14^ cells/ml) was diluted to various degrees using PBS as a diluent. The procedure thus yielded two ways of obtaining equal number of the two organisms but with different initial concentrations. Each of these tubes were used to obtain optical density (1 ml) using spectrophotometer (at 600 nm, Model V530, Jasco corporation, Japan), CFU using spread plate technique (0.1 ml) by incubating for 24 h at 37°C and rate of decoloration using MBRT. In the spread plate technique, at least 4 different dilutions were used to obtain an average colony forming units. The samples from the dilutions were also used to obtain individual colonies of *E. coli* and *B. subtilis*. Differential agar plates were used to differentiate the two organisms. MacConkey agar plate was used to quantify *E. coli*, while phenyl ethyl alcohol Hiveg™ agar was used to quantify *B. subtilis*. Thus, the MBRT slope was related to the OD of mixed culture, CFU of mixed culture, CFU of *E. coli* in mixed culture and CFU of *B. subtilis* in mixed culture. MBRT could be linearly related to all of the above measurements. However, it should be noted that for measurements less than 1000 cells, the sensitivity of MBRT reduced resulting in a nonlinear relationship (below an OD of 0.05). The sample growth rate was evaluated by obtaining the slope of linear profile using the plot of CFU in the log scale. Similarly, slope of the profile obtained using the log of the MBRT slope also yielded the specific growth rate since the slope of MBRT was directly related to the CFU.

### Growth experiments

*E. coli* and *B. subtilis* were separately grown in shake flask at 240 rpm and at 37°C. The cells (1% v/v) of each of the organism were inoculated into another 100 ml sterile Luria broth and grown for 25 h. MBRT, total CFU, CFU of *E. coli* and CFU of *B. subtilis* were evaluated at regular intervals.

## Results

**Figure 1:**
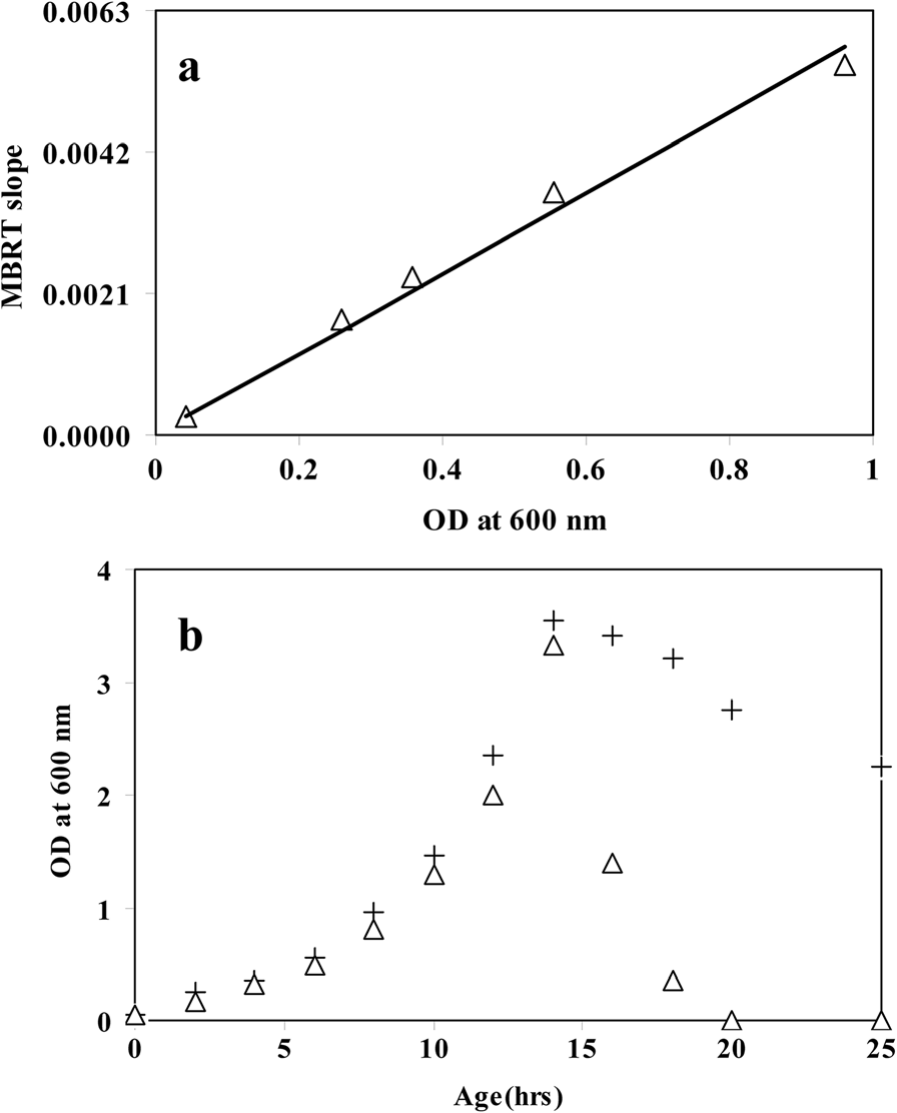
**Standard curve relating Optical Density and the Slope of Methylene Blue (MB) reduction in the mixed culture of*E. coli*and*B. subtilis*. (a)** Linear relationship between optical density and MBRT slope. Symbol open triangle represents experimental points. Solid line represents best fit with R^2^ = 0.987. Best-fit equation is given by Y = 0.0063 X. **(b)** Variation of OD at different time points during the growth of the mixed culture. Symbol open triangle represents equivalent OD at 600 nm as determined by MBRT; plus or positive symbol represents actual OD measured.

Firstly, we established a linear relationship between the optical density measurement (OD) and the MBRT slope obtained from a mixed culture of *E. coli* and *B. subtilis*. Figure 1a shows that the OD and MBRT slope demonstrated a linear relationship. It should be noted that the cells used for establishing the linearity were obtained from the exponential growth phase. This ensures that all the cells were metabolic active in the broth. Thus, an experiment was performed where both *E. coli* and *B. subtilis* were inoculated in Luria broth. Measurement of both OD and MBRT were performed during the course of the experiment. Figure 1b shows the profiles of OD and the equivalent OD obtained from MBRT using the linearity from Figure 1a. The OD measured from the spectrophotometer matched the MBRT until the end of the exponential growth phase. Beyond which the two ODs do not match, since MBRT is related to the metabolic active cells, while OD measures the turbidity. It was observed that by the end of 20 h, the OD equivalent obtained from MBRT was very small (indicating less than 1000 metabolic active cells).

**Figure 2:**
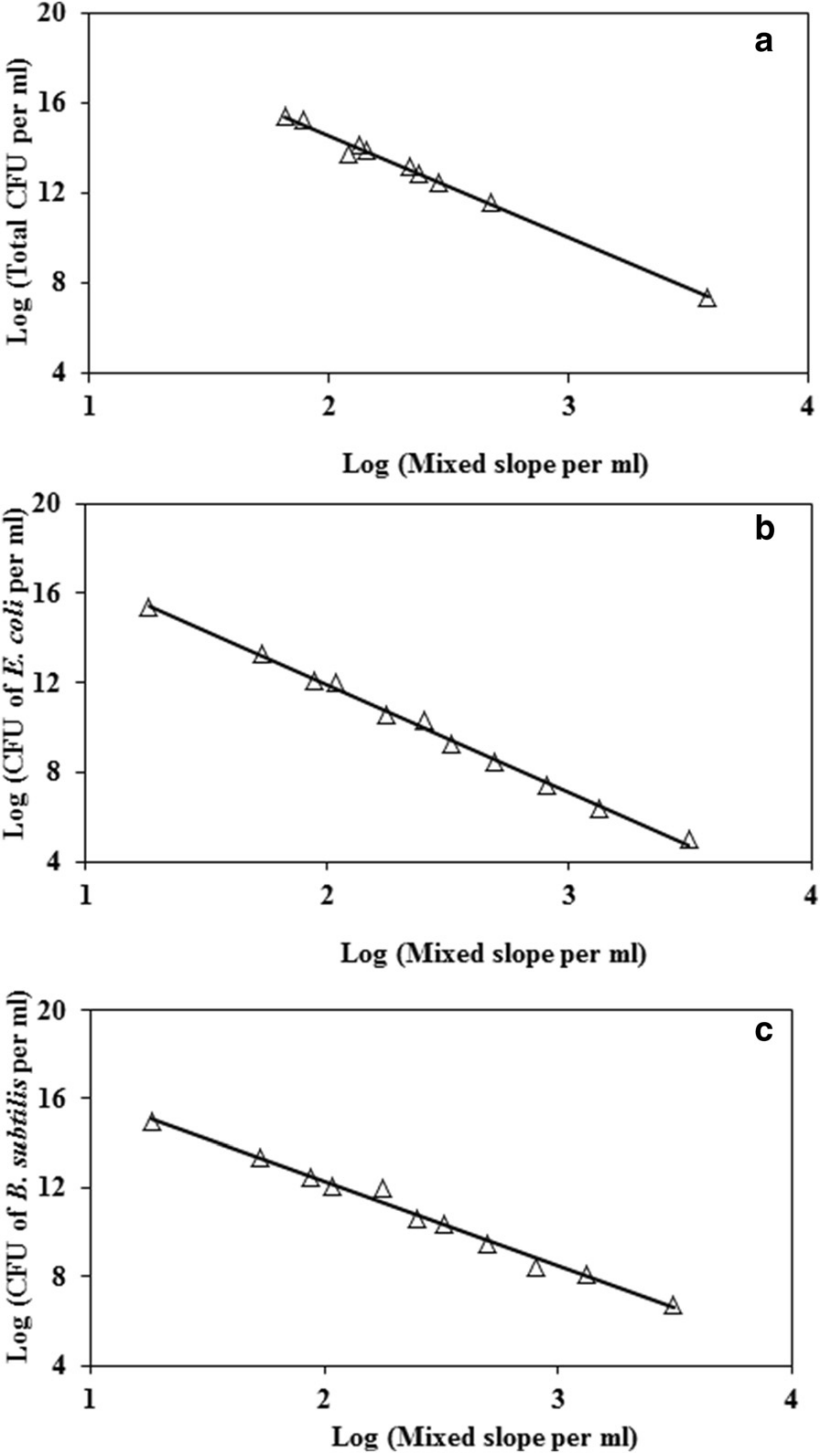
**Standard curves relating log of CFU with respect to the norm of the Log of slope of MB reduction. (a)** Total metabolic active cell count versus MBRT slope. Symbol open triangle represents experimental points. In this case, Log (MBRT slope) and Log (CFU) were fitted as a straight line. Solid line represents best fit with R^2^ = 0.99. Best-fit equation is given by Y = −4.7* X + 24. **(b)** Total metabolic active *E. coli* count versus MBRT slope for the mixed culture. Symbol open triangle represents experimental points. In this case, Log (CFU) and Log (MBRT slope) were fitted as a straight line. Solid line represents best fit with R^2^ = 0.99. Best-fit equation is given by Y = −4.8* X + 21.5. **(c)** Total metabolic active *B. subtilis* count versus MBRT slope for the mixed culture. Symbol open triangle represents experimental points. In this case, Log (CFU) and Log (MBRT slope) were fitted as a straight line. Solid line represents best fit with R^2^ = 0.98. Best-fit equation is given by Y = −4.2* X + 19.882.

A standard curve between log of MBRT slope and log of total CFU was obtained by considering mixed cultures of *E. coli* and *B. subtilis* (See Figure 2a). Similarly, the standard curve of log MBRT slope for mixed cultures versus log of CFU of *E. coli* cells alone (See Figure 2b) and log of MBRT slope for mixed cultures versus log of CFU of *B. subtilis* cells alone (See Figure 2c) were also obtained. It was interesting to note that the MBRT slope for the mixed culture was linearly related to not only the CFU of mixed culture but also to the individual CFU of *E. coli* and *B. subtilis* cells. Both microorganisms were diluted and spread onto selective solid media for determination of individual CFUs and to correlate these to MBRT mixed slope as discussed in Methylene blue dye reduction test in Materials and methods. Thus, the slope of MBRT obtained from a mixed culture can be directly used to estimate not only the total CFU but also the individual CFUs. These standard curves were used to determine the CFUs during the growth of *E. coli* and *B. subtilis* in a mixed culture. Once standard curves for individual microorganism are available relating MBRT slope and plate technique, and then there is no need to use the plate method in future experiments.

**Figure 3:**
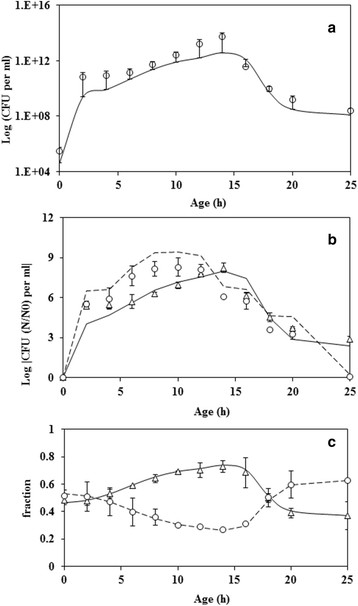
**Comparison of CFU obtained through MBRT and that obtained through direct measurement from agar plate method. (a)** Symbol open circle represents data obtained from agar plate method. Continuous line represents equivalent CFU determined by MBRT. Maximum error in the estimation was 18%. **(b)** Effect of different initial concentration of total cell count on the growth of mixed culture. Solid line (CFU equivalent from MBRT) and the symbol open triangle (data from agar plate method) are for an initial cell concentration of 10^4^ cells/ml. Dashed line (CFU equivalent from MBRT) and the symbol open circle (data from agar plate method) is for an initial cell concentration of 10^2^ cells/ml. It can be seen that the MBRT is able to predict the effect of initial cell concentration. **(c)** Fraction of individual species to total metabolic active cell count for the mixed culture of *E. coli* and *B. subtilis*. Symbol open triangle and open circle represent *E. coli* and *B. subtilis* respectively.

Figure 3a shows the estimate of CFU obtained from measuring the slope of MB reduction and using the standard curve (Figure 2a). The figure also compares the estimate with the experimental measurement using agar plates. MBRT could estimate the total CFUs in a mixed culture. The maximum error observed was about 18%. The slope of MB reduction was also used to estimate the individual CFU’s for *E. coli* and *B. subtilis* (using Figure 2b and 2c, respectively). Figure 3b shows the time profile of the total number of metabolic active cells obtained using MBRT at various time points for two different initial concentration of total cell count with almost equal number of the two cells. The figure also compares the normalized total number of cells (N/N0) with that obtained through the plate count method. In the case of an initial cell count of 10^4^ cells/ml (solid line), the cell number increased to 10^9^ cells/ml in about 14 h. This resulted in a growth rate of 0.65 h^−1^ which could be obtained directly through the measurement using MBRT. In the case of an initial cell count of 10^2^ cells/ml (dashed line), the cell number increased to 10^9^ cells/ml in about 12 h, indicating a faster growth rate at lower initial concentration of cells. The cell number decreased to 10^5^ cells/ml at t = 25 h. This resulted in a growth rate of 0.70 h^−1^. After a stationary phase of 5 h, the cell count decreased to 10^5^ cells/ml at t = 25 h, which was the same as observed in the case of initial concentration of 10^4^ cells/ml. The mixed culture resulted in a death rate constant of 0.56 h^−1^. Figure 3c shows the comparison of the estimates of CFU obtained from MBRT and from agar plates for an initial metabolic active count of 10^4^ cells/ml. The data is presented as a fraction of total CFU. It is clear that the MBRT of a mixed culture could predict the CFU of individual microorganism. Although the initial cell count for *E. coli* and *B. subtilis* were almost equal, *E. coli* demonstrated a higher growth rate as compared to *B. subtilis*. The fraction of *E. coli* in the population was higher upto 17 h and later *B. subtilis* had a higher fraction of cells. It should be noted that since the total cells count increased with time, the count of both *E. coli* and *B. subtilis* increased with time, which was correlated well by MBRT.

**Figure 4:**
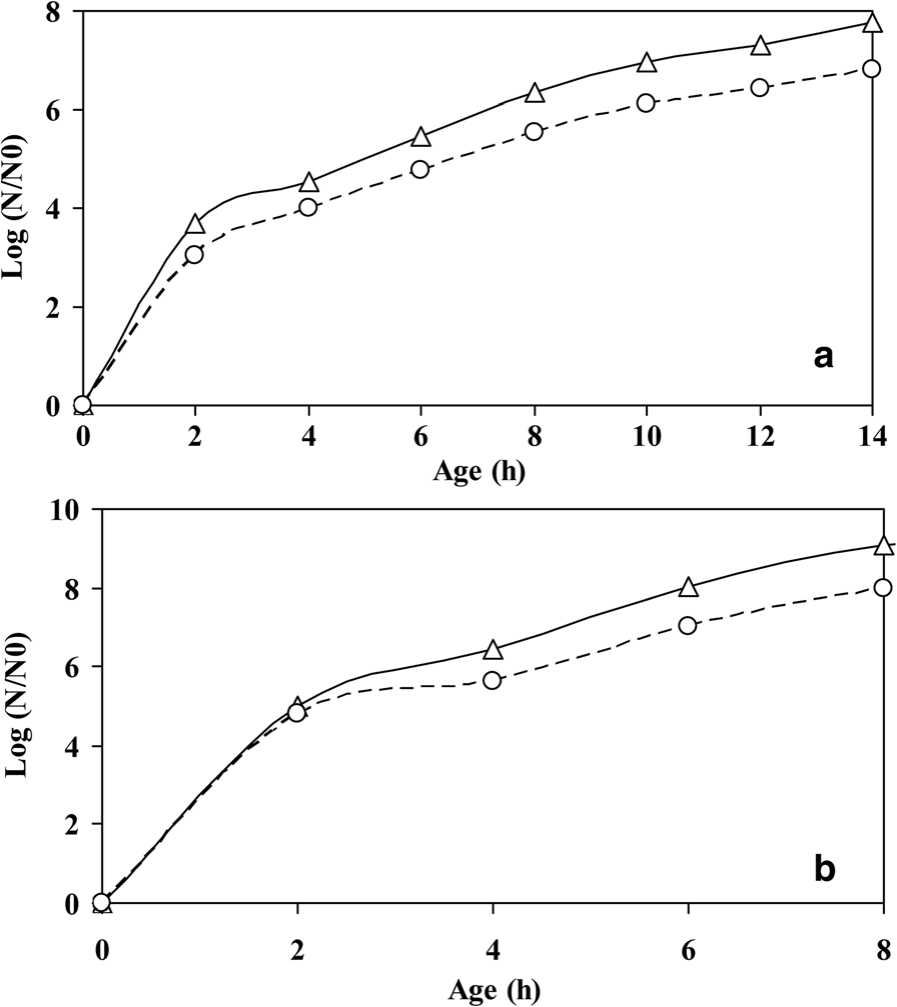
**Comparison of CFU obtained through MBRT and that obtained through direct measurement from agar plate method for*E. coli*and*B. subtilis*. (a)** Metabolic active cell count of *E. coli* (open triangles) and *B. subtilis* (open circles) for initial total metabolic active count of 10^4^ cells. **(b)** Metabolic active cell count of *E. coli* (open triangles) and *B. subtilis* (open circles) for initial total metabolic active cell count of 10^2^ cells.

Figure 4 shows the metabolic active cell count of *E. coli* and *B. subtilis* in the mixed culture. Figure 4a represents the metabolic active cell count of only *E. coli* (solid line) as obtained from MBRT, with a growth rate equivalent of 0.31 h^−1^ (for an initial total metabolic active cell count of 10^4^ cells/ml). Similarly, the metabolic active cell count for *B. subtilis* (dashed line) with a growth rate of 0.35 h^−1^. When the initial cell count was kept at 10^2^ cells/ml, the growth rate of *E. coli* alone in the mixed culture was 0.34 h^−1^ (see solid line Figure 4b) and 0.37 h^−1^ for the *B. subtilis*. All mixed culture experiments were performed in triplicate.

**Figure 5:**
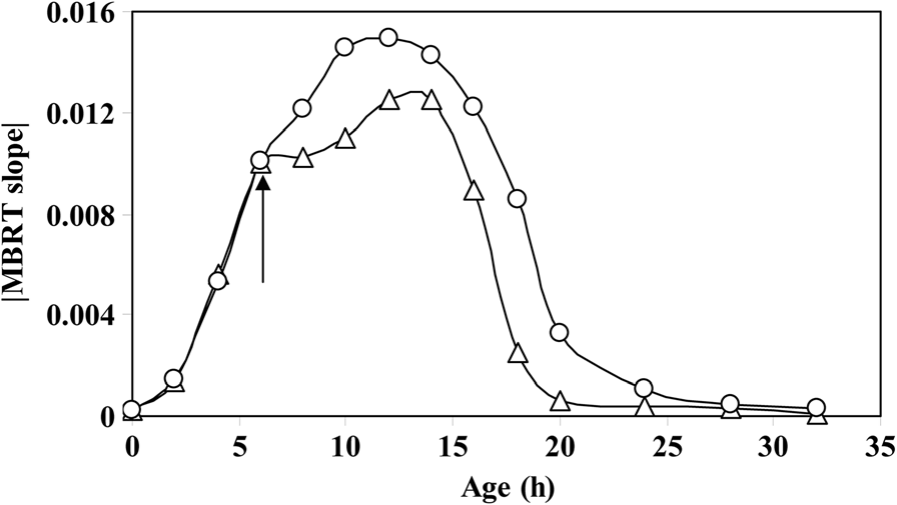
**Comparison of the slope of methylene blue decoloration of growth of*B. subtilis*(open triangle) and introduction of*E. coli*at t = 6 h during the growth of*B. subtilis*(open circle).** It is clearly seen that the deviation at 6 h in the value of the slope indicated that the cell number had indeed increased. This method can be used to monitor cell growth of pure cultures. The arrow shows the addition of *E. coli* in the growth of *B. subtilis* as a contaminant.

Further, in a 100 ml flask where only *B. subtilis* had grown (approximately 10^3^ cells), around 500 cells of *E. coli* were introduced at t = 6 h. This experiment was conducted to determine the effect of a contaminant on the MBRT slope. MBRT was used to measure the slope of decoloration in the two flasks. Figure 5 shows the value of slope of decoloration with time for the two cases. It can be seen that the slope of MBRT deviated at the point of inoculation of *E. coli* (at t = 6 h) where OD change due to the addition of *E. coli* is negligible. The increase in the slope indicated that higher number of metabolic active cells were present beyond 6 h. There is a difference in MBRT slope before and after addition of *E. coli* cells but there is not any difference in OD change. Thus, MBRT can be used to monitor contamination of a fermentation process without making plate count. Thus, the method can be used for online measurement of numbers of metabolically active cells indicating contamination in case of deviation from the normal profile.

## Discussion

**Figure 6:**
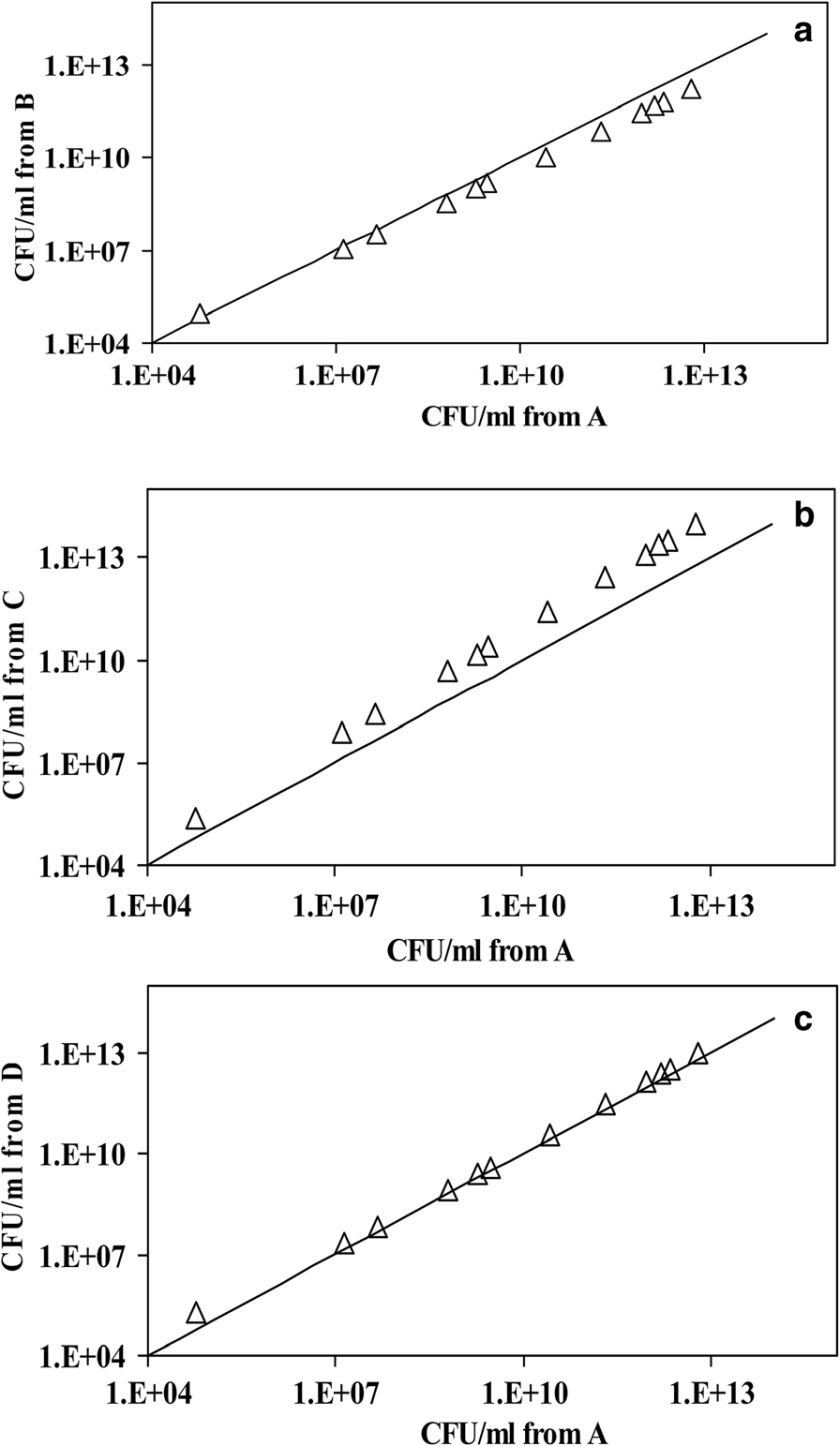
**Comparison of CFU obtained through different protocols as described in the text. (a)** Comparison the CFU obtained using protocol ‘A’ and ‘B’. **(b)** Comparison the CFU obtained using protocol ‘A’ and ‘C’. **(c)** Comparison the CFU obtained using protocol ‘A’ and ‘D’. Protocol A: CFU was obtained using MBRT and the standard curve relating slope of MB reduction and CFU of mixed culture. Protocol B: Individual CFU of *E. coli* and *B. subtilis* were obtained using the slope of MB reduction of mixed culture and these CFU’s were added to obtain total CFU count. Protocol C: CFU’s of *E. coli* and *B. subtilis* were used to obtain the MBRT slopes from pure cultures and the slopes were added to obtain the net MBRT slope for a mixed culture. Protocol D: Measurement of the slope of MB reduction in a mixed culture and used the slope directly in the standard curve of the pure cultures to obtain the CFU’s of *E. coli* and *B. subtilis*, which was then added to yield the total CFU.

Four different methodologies were used to determine the CFU in the mixed culture. Firstly, CFU was obtained using MBRT and the standard curve relating slope of MB reduction and CFU of mixed culture which was termed as protocol ‘A’. Secondly, the individual CFU of *E. coli* and *B. subtilis* were obtained using the slope of MB reduction of mixed culture and these CFUs were added to obtain total CFU count, termed as protocol ‘B’. Thirdly, the CFUs of *E. coli* and *B. subtilis* were used to obtain the MBRT slopes from pure cultures and the slopes were added to obtain the net MBRT slope for the mixed culture. This was further used to estimate total CFU and was termed as protocol ‘C’. The fourth method used the measurement of the slope of MB reduction in a mixed culture and used the slope directly in the standard curve of the pure cultures to obtain the CFUs of *E. coli* and *B. subtilis*, which was then added to yield the total CFU. Figure 6 shows the comparison of the estimate of CFU obtained from the above protocol.

It is clear from the figure that all the four protocol can be used to estimate the CFUs within the error (Maximum 12%). This indicated that the standard curves from pure culture could be used to obtain the total CFU (protocol ‘D’), using slope of MB reduction slope from a mixed culture, demonstrating the utility of the methodology. The reason for the applicability of MBRT slope of mixed culture using standard curves of pure culture may be due to the dominance of one microorganism over the other at any given time (See Figure 3a).

The above experiments demonstrated that MBRT can be used to quantify not only the total cell count but also the individual CFUs of different organisms in the mixed culture (Nandy et al. [2007], Nandy et al. [2008], Nandy and Venkatesh [2008], Nandy and Venkatesh [2010]) with approximately same known initial cell concentration. Thus, the next step was to use the methodology to monitor fermentations which is exhibited in Figure 5.

We have presented here a methodology for quantification of metabolic active cell in mixed culture using Methylene Blue dye Reduction Test (MBRT). The slope of the rate of reduction of Methylene Blue was correlated to the metabolically active cell count. This was compared to CFU obtained using agar plate method to demonstrate the utility of the test. Further, starting from the approximately same initial cell concentration of different species, the MBRT was also used to quantify the metabolically active cell count for the individual cells in a mixed culture, of *E. coli* and *B. subtilis*. It was noted that the standard curve relating the slope of MB reduction and CFU for the individual cells could be used to measure the metabolic activity of each species.. The individual growth rates were evaluated at the end of the growth experiment itself as MBRT yielded the metabolic active cell count in less than 6 minutes at each time point. Thus, by the end of the exponential growth rate (i.e. about 12 h in case of initial cell count of 10^2^ cells/ml) the individual growth rate could also be estimated, whereas in case of the agar plate method, one needs to streak the cells on differential plates for *E. coli* and *B. subtilis* at the end of 8 h and obtain the cell count only after 24-30 h of incubation. This demonstrated the strength of the method reported here. Initial known cell concentration is only the limitation of this method. It is possible to add an unknown species with initial cell concentration in a known culture concentration; then one could calculate the number of cell by MBRT but would need to take MBRT slope before and after addition. The difference in slope may exhibit the addition of cell concentration in the culture. It will be a challenge for future work to estimate unknown individual initial cell concentrations in a mixed culture.

Further, we have also demonstrated that the slope of decoloration of methylene blue can be used to monitor fermentations online. Since the slope obtained from MBRT is indicative of the total metabolic active cell, a standard profile for normal operation can be established. During operation deviations from the normal profile may indicate contamination or abnormal behavior. Since the metabolic active cell count increased in case of contamination, the MBRT slope would be higher than the MBRT slope for normal operation. If the MBRT slope obtained during monitoring is less than that of the normal profile, abnormalities such as failure in temperature or pH control, oxygen limitation or inhibitors in the medium, may be the cause for decrease in metabolic active cell. Thus, MBRT can be used for fault diagnosis through online measurement of slope of MB decoloration.

The evaluation of the total metabolic active cell count and the individual metabolic active cell counts starting with an equal cell numbers of different species present in a mixed culture offers a quantification which would be helpful in designing, operating and controlling processes using mixed cultures. Further, the evaluation of growth rates of the individual species during the growth of mixed culture in a process offers insights into the operation of such processes. Thus, the protocol developed here can be adapted to applications in processes involving mixed cultures.

## Competing interests

The authors declare that they have no competing interests.

## Authors’ contributions

SKN designed and carried out the experiments, analyse the data and write the manuscript. KVV helped to draft the manuscript. Both authors read and approved the final manuscript.
